# Impact of Digoxin Utilization on Gastrointestinal Bleeding in Patients With Continuous-Flow Left Ventricular Assist Devices: A Systematic Review and Meta-Analysis

**DOI:** 10.1097/MAT.0000000000002473

**Published:** 2025-05-18

**Authors:** Matteo Marchetti, Barbara Pitta Gros, Olivier Muller, Clémence Ferlay, Zied Ltaief, Matthias Kirsch, Anna Nowacka, Patrizio Pascale, Philippe Meyer, Patrick Yerly, Pierre Monney, Panagiotis Antiochos, Hicham Skali, Maja Cikes, Roger Hullin, Henri Lu

**Affiliations:** From the * Division of Cardiology, Lausanne University Hospital (CHUV), University of Lausanne (UNIL), Lausanne, Switzerland; † Division of Adult Intensive Care Medicine, Lausanne University Hospital (CHUV), University of Lausanne (UNIL), Lausanne, Switzerland; ‡ Division of Cardiac Surgery, Lausanne University Hospital (CHUV), University of Lausanne (UNIL), Lausanne, Switzerland; § Division of Cardiology, Geneva University Hospital, University of Geneva, Geneva, Switzerland; ¶ Division of Cardiovascular Medicine, Brigham and Women’s Hospital, Harvard Medical School, Boston, Massachusetts; ‖ Department of Cardiovascular Diseases, University of Zagreb School of Medicine, University Hospital Centre Zagreb, Zagreb, Croatia.

**Keywords:** digoxin, left ventricular assist device, gastrointestinal bleeding, angiodysplasia

## Abstract

Continuous-flow left ventricular assist devices (CF-LVADs) improve quality of life and survival in patients with advanced heart failure but are frequently complicated by gastrointestinal bleeding (GIB). Reduced pulsatile flow may induce mucosal hypoxia, upregulating factors such as hypoxia-inducible factor (HIF)-1α and triggering neo-angiogenesis, leading to the development of gastrointestinal angiodysplasias (GIADs), a common cause of GIB. Digoxin inhibits HIF-1α and may prevent GIAD development, although its impact on the incidence of GIB remains uncertain. This meta-analysis (PROSPERO ID: CRD42024626222) evaluated the association between digoxin use and GIB occurrence (primary outcome) in patients with CF-LVADs. Research articles including adults with CF-LVADs, comparing digoxin users *versus* nonusers were included. Overall, four studies were included (n = 14,917; age 55 ± 13 years, 21% female) with 2,742 patients in the digoxin group and 12,175 in the no-digoxin group. Continuous-flow left ventricular assist device was axial (HeartMate II) in 78% of cases and centrifugal (HeartMate 3/HeartWare) in 22%. Digoxin use was associated with a nonsignificant lower risk of GIB (hazard ratio [HR]: 0.70; 95% confidence interval [CI]: 0.49–1.01). However, regarding GIAD-related GIB, digoxin was associated with a significantly lower risk (HR: 0.33; 95% CI: 0.13–0.82). Among 14,917 patients with CF-LVADs, digoxin use was associated with a trend toward a lower risk of GIB and a lower risk of GIAD-related GIB.

Continuous-flow left ventricular assist devices (CF-LVADs) substantially improve quality of life and survival in selected patients with advanced heart failure.^1^ Despite these benefits, CF-LVADs are often associated with complications, particularly gastrointestinal bleeding (GIB), which affects up to 27% of patients.^2–4^ GIB remains a major cause of morbidity and hospitalizations in this population,^5,6^ with gastrointestinal angiodysplasias (GIADs), resulting from arteriovenous malformations, being a common underlying cause.^7^

From a pathophysiological perspective, the exact mechanism of this process remains unclear. A key contributing factor is likely the reduction or loss of pulsatile flow, which occurs with LVAD treatment. The latter contributes to the depletion of high-molecular-weight multimers of von Willebrand factor, enhances thrombin generation, and triggers inflammation, ultimately promoting bleeding and impairing angiogenesis.^8^ It has been postulated that these hemodynamic and vascular alterations may result in submucosal splanchnic hypoxia, triggering signaling cascades in intestinal mucosa and endothelial cells.^9^ This process involves the upregulation of angiogenic factors such as angiopoietin-1 (Ang-1), angiopoetin-2 (Ang-2), vascular endothelial growth factor (VEGF), and hypoxia-inducible factor (HIF)-1α and HIF-2α, which in turn leads to neo-angiogenesis and subsequent formation of GIADs.^10–15^

Digoxin has been shown to inhibit HIF-1α expression,^16^ potentially reducing the formation of GIADs and presenting therapeutic potential in patients with CF-LVADs.^12^ However, the effectiveness of digoxin in preventing GIB in this population remains unclear, as existing literature presents heterogeneous findings, and no prospective randomized clinical trials have been conducted to establish its efficacy. This uncertainty is further compounded by the need to balance its potential benefits against the side effects associated with its narrow therapeutic window. We performed this systematic review and meta-analysis to assess the association between digoxin utilization and GIB risk in patients supported by CF-LVADs.

## Methods

This systematic review with meta-analysis followed a prespecified study protocol, registered in the PROSPERO International Prospective Register of Systematic Reviews (under the title “Impact of Digoxin Utilization on Gastrointestinal Bleeding in Patients With Continuous-Flow Left Ventricular Assist Devices: A Systematic Review and Meta-Analysis,” identification number: CRD42024626222). This work adhered to the recommendations of the International Committee of Medical Journal Editors,^17^ and followed the Preferred Reporting Items for Systematic Reviews and Meta-Analyses (PRISMA) guidelines.^18^ Ethical approval was not required, as our meta-analysis was conducted at the study level using previously published data.

### Literature Research Strategy and Data Collection

A systematic review of all relevant studies was performed, covering the period from inception to November 30, 2024, using the PubMed/MEDLINE (Medical Literature Analysis and Retrieval System Online) and EMBASE (Excerpta Medica Database) platforms. The following research keywords were used: “left ventricular assist device,” “LVAD,” “mechanical circulatory support,” “digoxin,” “gastrointestinal bleeding,” “GI bleeding,” “GI bleed.” All original research articles including adult patients with CF-LVADs, comparing digoxin users *versus* nonusers were assessed for eligibility, without any restriction or filter.

Studies were included in the analysis if they met the following criteria: original research articles published in English, comparison of digoxin users *versus* nonusers, availability of data on baseline population characteristics, and documentation of GIB events. Clinical case reports, review articles, meta-analyses, and nonhuman research were excluded. Two authors (M.M. and B.P.G.) independently assessed study eligibility and performed data extraction, and any discrepancy was resolved through consensus with the help of a third author (H.L.).The primary outcome was GIB occurrence, and the secondary outcome was GIB likely attributable to GIAD (GIB-GIAD). The quality of each included study was independently evaluated by two authors (M.M. and B.P.G.) using the Newcastle−Ottawa scale and the GRADE (Grading of Recommendations Assessment, Development, and Evaluation) approach.^19,20^

### Statistical Analyses

For each study, baseline patient characteristics were extracted, with continuous variables summarized as means ± standard deviations (SDs) or medians (interquartile ranges [IQRs]), and categorical variables reported as counts with percentages.

A random-effects model was used to account for population diversity and methodological variation among studies, and heterogeneity across studies was assessed using the Cochran *Q* statistic and the Higgins and Thompson Heterogeneity test (*I^2^*).^21^ The *I*² statistic was used to quantify the proportion of variation across studies attributable to heterogeneity rather than chance, with thresholds defined as follows: less than 25% indicating low heterogeneity, 25–50% (moderate heterogeneity), and greater than 50% (high heterogeneity). Hazard ratios (HRs) and their corresponding 95% confidence intervals (CIs) were extracted from each study and log-transformed before the meta-analysis. Values adjusted for baseline characteristics were used whenever available.

The meta-analysis was performed on the natural logarithmic HR scale, results were then exponentiated and reported on the original HR scale. A sensitivity analysis excluding the study with the highest weight was performed, to assess the consistency of the results in the remaining studies.^22^ All *p* values were two-sided and a value less than 0.05 was considered statistically significant. Analyses were carried out using Stata (version 18, StataCorp LLC, College Station, TX).

## Results

### Study Selection and Quality Assessment

**Figure 1.**
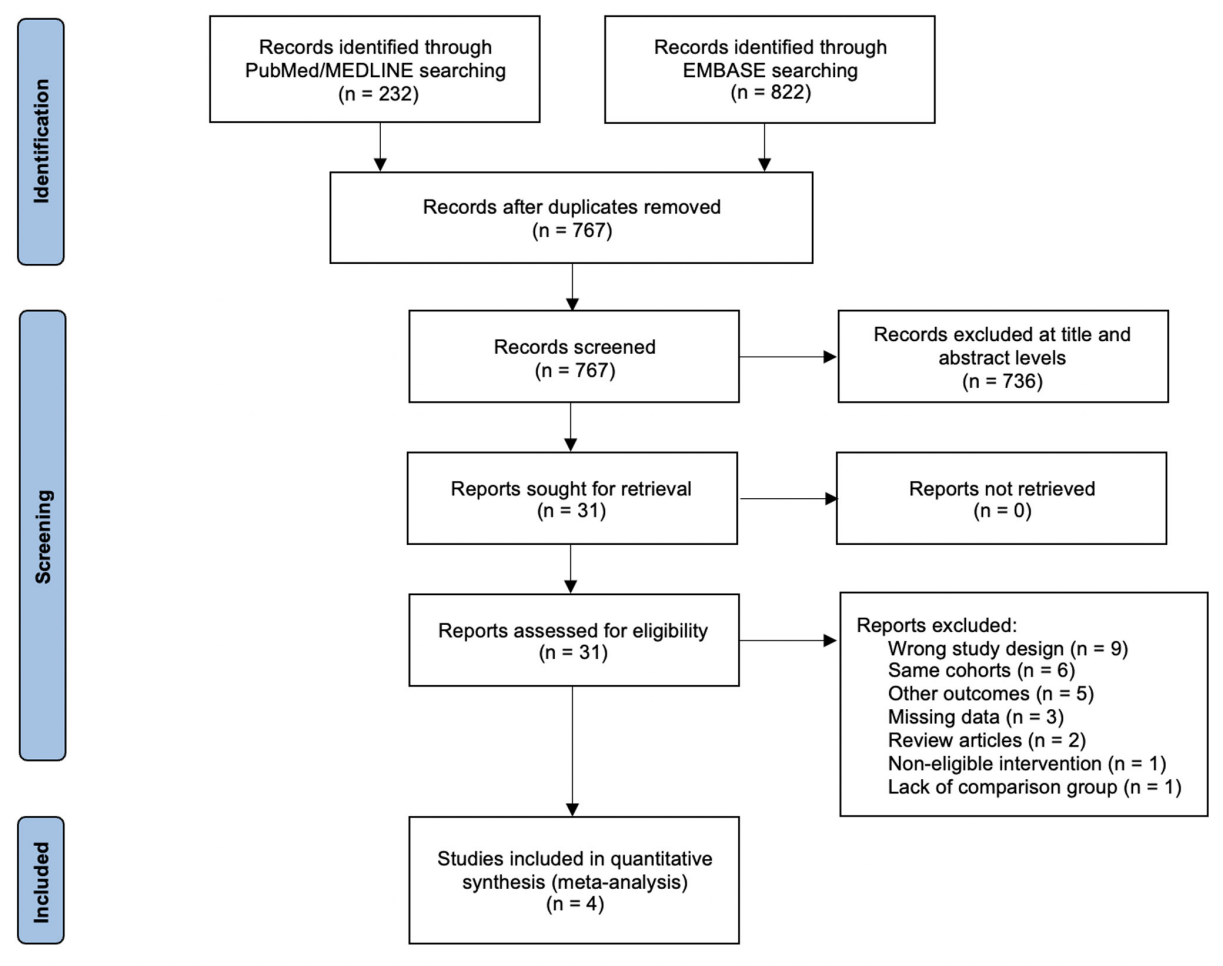
PRISMA flow diagram. EMBASE, Excerpta Medica Database; MEDLINE, Medical Literature Analysis and Retrieval System Online; PRISMA, Preferred Reporting Items for Systematic Reviews and Meta-Analyses.

A total of 1,054 references were initially identified, with 287 duplicates removed and 736 references deemed irrelevant during the title and abstract screening stage. Of the remaining 31 studies, 4 met the inclusion criteria (Figure 1). All included studies were retrospective in nature, with one study reporting data from the multicenter Interagency Registry for Mechanically Assisted Circulatory Support (INTERMACS).^23^

Based on the Newcastle−Ottawa Scale and the GRADE approach, three studies were rated as having high quality with a low risk of bias,^12,24,25^ whereas one study was rated as having moderate risk of bias and quality (Tables 1 and 2, Supplemental Digital Content, https://links.lww.com/ASAIO/B525).^23^

### Definitions of Outcomes and Study Groups

Gastrointestinal bleeding was similarly defined in three studies, as clinical evidence of bleeding (hematochezia, hematemesis, melena, or positive fecal-occult blood test), along with a hemoglobin decrease of at least 1 g/dl.^12,24,25^ In contrast, Jennings *et al.*^23^ defined GIB as suspected internal or external bleeding that resulted in one or more of the following: (1) death, (2) reoperation, (3) hospitalization, (4) red blood cell transfusion.

Gastrointestinal bleeding-GIAD was defined in two studies.^12,24^ Vukelic *et al.*^12^ defined GIB-GIAD as the visualization of angiodysplasia lesions during endoscopic procedures or video capsule in the absence of any other bleeding source. Episodes suspected to be GIB-GIAD were defined as cases where no bleeding source was identified despite thorough endoscopic evaluations, including video capsule.^12^ El Rafei *et al.*^24^ defined GIB-GIAD as cases in which GIAD lesions were identified, no alternative source of bleeding was found during the index bleed, or patients who experienced more than two episodes of GIB while on LVAD support.

The definition of digoxin use varied among studies. Vukelic *et al.*^12^ classified patients in the digoxin group if they received digoxin for at least seven days after LVAD implantation and were receiving digoxin at discharge. Jennings *et al.*^23^ defined digoxin use based on its prescription status three months after CF-LVAD implantation. El Rafei *et al*.^24^ categorized patients in the digoxin group if digoxin was prescribed either at discharge or within 3 months post-discharge, following CF-LVAD implantation. In the last study, digoxin use was defined as the use of digoxin for at least 30 days after CF-LVAD implantation (Table 3, Supplemental Digital Content, https://links.lww.com/ASAIO/B525).^25^

### Baseline Characteristics

A total of 14,917 patients (mean age 55 ± 13 years, 21% female, 45% with ischemic heart disease) were included, with 2,742 in the digoxin group and 12,175 in the no-digoxin group (Table 1). All patients were on vitamin K antagonist (VKA) anticoagulation, with an international normalized ratio (INR) target of 2–3, alongside antiplatelet therapy (primarily aspirin) at varying doses (81–325 mg/day for aspirin). Continuous-flow left ventricular assist device support was axial (HeartMate II) in 78% of patients and centrifugal (HeartMate 3 or HeartWare) in 22% (Tables 1 and 2).

**Table 1.** Patient Baseline Characteristics

Reference	Number of Patients	Type of CF-LVAD, n (%)	Age (Years)	Women	Creatinine (mg/dl)	IHD	INTERMACS Profile	Diabetes	LVEF (%)
Vukelic *et al.*^12^	O: 199	O: 172 (86) HM II, 27 (14) HW	O: 55 ± 13	O: 48 (24)	O: 1.0 (0.8–1.4)	O: 80 (40)	O: N.A.	O: 77 (39)	O: 20 ± 7
	D+: 64	D+: 51 (81) HM II, 13 (19) HW	D+: 54 ± 13	D+: 15 (23)	D+: 1.0 (0.8–1.4)	D+: 22 (34)	D+: N.A.	D+: 23 (36)	D+: 19 ± 6
	D−: 135	D−: 119 (88) HM II, 16 (12) HW	D−: 55 ± 13	D−: 33 (24)	D−: 1.1 (0.9–1.5)	D−: 58 (43)	D−: N.A.	D−: 54 (40)	D−: 20 ± 7
Jennings *et al.*^23^	O: 13,723	O: 2,986 (22) centrifugal and 10,746 (78) axial LVADs	O: 55 ± 13	O: 2,850 (21)	O: 1.4 ± 0.7	O: 6,026 (43)	O: 1, 2,080 (15); 2, 4,891 (36); 3, 4,472 (33); 4–7, 2,235 (16)	O: 507 (4)	O: N.A.
	D+: 2,321	D+: N.A.	D+: N.A.	D+: N.A.	D+: N.A.	D+: N.A.	D+: N.A.	D+: N.A.	D+: N.A.
	D–: 11,402	D−: N.A.	D−: N.A.	D−: N.A.	D−: N.A.	D−: N.A.	D−: N.A.	D−: N.A.	D−: N.A.
El Rafei *et al.*^24^	O: 649	O: 509 (78) HM II, 82 (13) HM 3, 58 (9) HW	O: 57 ± 14	O: 520 (20)	O: 1.2 (1.0–1.6)	O: 290 (45)	O: 1, 91 (14); 2–3, 327 (50); 4+, 231 (36)	O: 272 (42)	O: N.A.
	D+: 213	D+: 169 (79) HM II, 18 (8) HW, 27 (12) HM 3	D+: 57 ± 14	D+: 43 (20)	D+: 1.2 (0.9–1.4)	D+: 107 (50)	D+: 1, 36 (17); 2–3, 121 (57); 4+, 56 (26)	D+: 97 (46)	D+: N.A.
	D−: 436	D−: 340 (78) HM II, 40 (9) HW, 56 (13) HM 3	D−: 58 ± 13	D−: 87 (20)	D−: 1.2 (1.0–1.7)	2 D−: 52 (58)	D−: 1, 55 (13); 2–3, 206 (47); 4+, 175 (40)	D−: 175 (40)	D−: N.A.
Abbasi *et al.*^25^	O: 346	O: 219 (64) HM II, 102 (29) HM 3, 25 (7) HW	O: 56 ± 13	O: 79 (23)	O: 1.4 ± 0.5	O: 181 (52)	O: 1, 63 (18); 2, 103 (30); 3, 106 (31); 4, 71 (21); 5, 1 (0)	O: 133 (38)	O: 18 ± 9
	D+: 144	D+: 21 (15) HM 3, 113 (78) HM II, 10 (7) HW	D+: 55 ± 13	D+: 29 (20)	D+: 1.3 ± 0.4	D+: 71 (49)	D+: 1, 31 (22); 2, 40 (28); 3, 45 (31); 4, 27 (19); 5, 0 (0)	D+: 58 (40)	D+: 18 ± 10
	D−: 202	D−: 80 (40) HM 3, 107 (53) HM II, 15 (7) HW	D−: 57 ± 13	D−: 49 (24)	D−: 1.4 ± 0.5	D−: 110 (54)	D−: 1, 32 (16); 2, 63 (31); 3, 61 (30); 4, 44 (22); 5, 1 (1)	D−: 75 (37)	D−: 19 ± 9

Results are reported as n (%), means ± standard deviations, or medians (interquartile range).

CF-LVAD, continuous-flow left ventricular assist device; D+, digoxin group; D−, no-digoxin group; HM, HeartMate; HW, HeartWare; IHD, ischemic heart disease; INTERMACS, Interagency Registry for Mechanically Assisted Circulatory Support; LVAD, left ventricular assist device; LVEF, left ventricular ejection fraction; N.A., not available; O, overall.

**Table 2.** Included Studies and Outcomes

Reference	Number of Patients	Antithrombotic Medication	GIB Occurrence
Vukelic *et al*.^12^	O: 199	O: VKA	O: 55 (27)
	D+: 64	D+: AAS in 56 (88), clopi in 2 (3)	D+: 10 (16)
	D−: 135	D−: AAS in 121 (90), clopi in 4 (3)	D−: 45 (33)
Jennings *et al*.^23^	O: 13,723	O: VKA + antiplatelet therapy	O: 2,674 (19)
	D+: 2,321	D+: N.A.	D+: 427 (18)
	D–: 11,402	D−: N.A.	D−: 2,247 (20)
El Rafei *et al.*^24^	O: 649	O: VKA + AAS at variable doses depending on type of LVAD (81 mg/d for HM II and HM 3 *vs.* 325 mg/day for HW)	O: N.A.
	D+: 213	D+: N.A.	D+: N.A.
	D−: 436	D−: N.A.	D−: N.A.
Abbasi *et al*.^25^	O: 346	O: VKA + AAS at variable doses depending on type of LVAD (81 mg/day for HM 3, 325 mg/day for HW, and 81 mg/day + dipyridamole for 3 months for HM II)	O: 1 GIB in 77 (22) and ≥ 2 GIB in 38 (11)
	D+: 144	D+: N.A.	D+: 22 (15) suffered ≥ 1 GIB
	D−: 202	D−: N.A.	D−: 53 (26) suffered ≥ 1 GIB

Results are reported as n (%), means ± standard deviations, or medians (interquartile range).

AAS, acetylsalicylic acid; CF-LVAD, continuous-flow left ventricular assist device; Clopi, clopidogrel; D+, digoxin group; D−, no-digoxin group; GIB, gastrointestinal bleeding; HM, HeartMate; HW, HeartWare; LVAD, left ventricular assist device; N.A., not available; O, overall; VKA, vitamin K antagonist.

### Digoxin Use and Gastrointestinal Bleeding Occurrence

**Figure 2.**
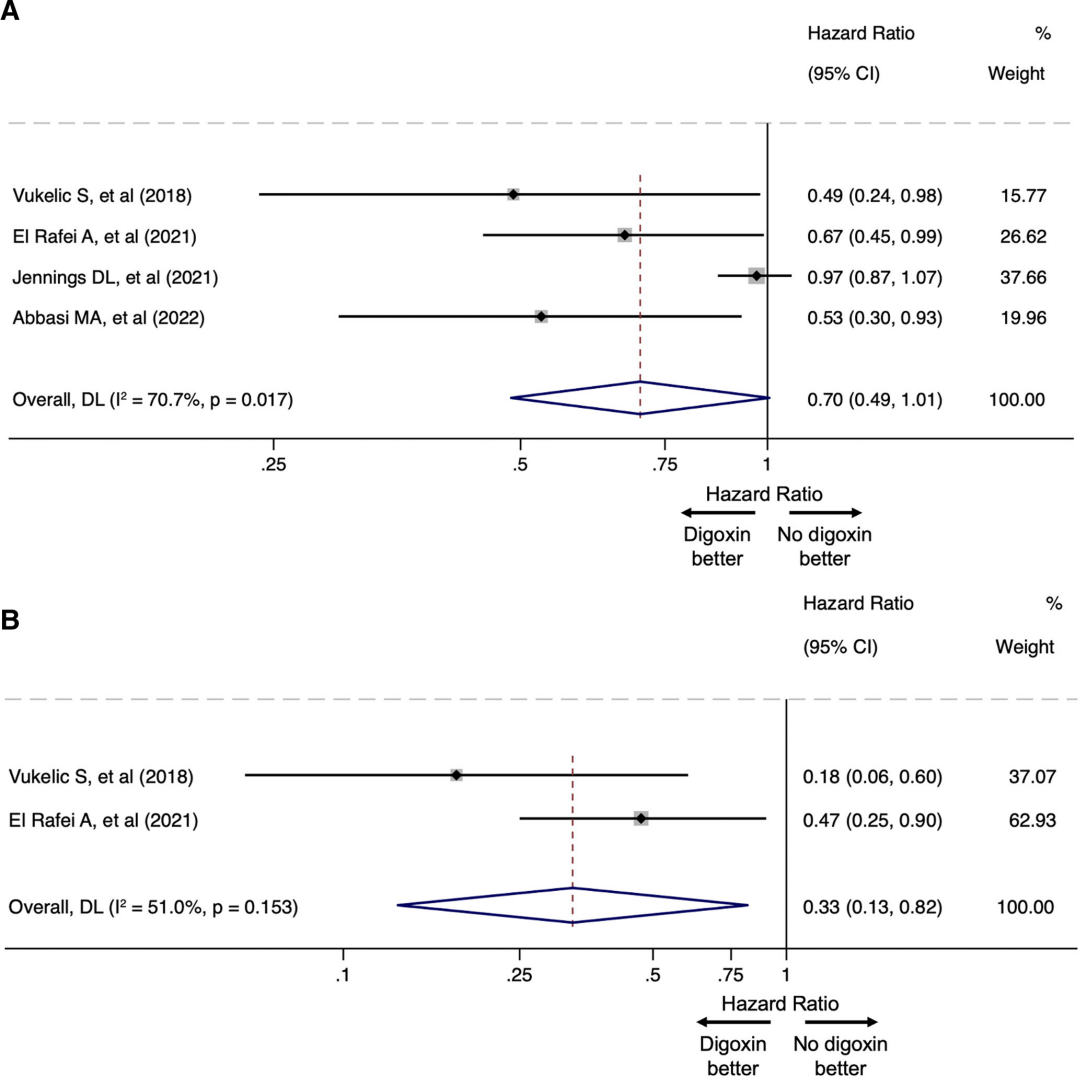
Forrest plots comparing (**A**) the risk of GIB between the digoxin and no-digoxin groups, (**B**) the risk of GIAD-related GIB between the digoxin and no-digoxin groups, among patients supported with CF-LVADs. CF-LVAD, continuous-flow left ventricular assist device; CI, confidence interval; GIAD, gastrointestinal angiodysplasia; GIB, gastrointestinal bleeding.

**Figure 3.**
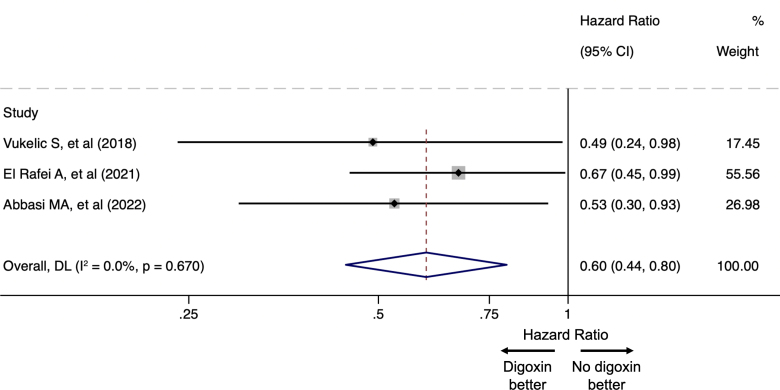
Forrest plot comparing the risk of GIB between the digoxin and no-digoxin groups among patients supported with CF-LVADs, excluding the study with the highest weight (Jennings *et al.*^23^). CI, confidence interval; CF-LVAD, continuous-flow left ventricular assist device; GIB, gastrointestinal bleeding.

Patients were followed for up to 24 months after CF-LVAD implantation in three studies.^23–25^ In the last one, the median follow-up duration was 10 months.^12^ Three studies reported the raw number of GIB events, with a total of 459 (16.7%) GIB events in the digoxin group and 2,345 (19.3%) in the no-digoxin group (GIB rate in the overall population: 18.8%).^12,23,25^ Overall, based on the results of all four studies, digoxin use was associated with an HR of 0.70 (95% CI: 0.49–1.01, *I*^2^: 70.7%) regarding the occurrence of GIB event (Figure 2A). After exclusion of the study with the highest weight (38%; Jennings *et al.*^23^), digoxin use was associated with an HR of 0.60 (95% CI: 0.44–0.80, *I*^2^: 0%) regarding GIB risk (Figure 3).

Two studies reported data on patients with GIB-GIAD: Vukelic *et al*.^12^ reported an occurrence rate of 10% among patients in the digoxin group, compared to 39% in the no-digoxin group. El Rafei *et al.*^24^ reported an adjusted incidence rate ratio of 0.47 (95% CI: 0.25–0.90, *p* = 0.022) for GIB-GIAD in the digoxin group compared to the no-digoxin group. Overall, digoxin use was associated with an HR of 0.33 (95% CI: 0.13–0.82; *I*^2^: 51.0%) for the occurrence of GIB-GIAD (Figure 2B).

None of the included studies reported exact digoxin dosing, however one study did compare median digoxin levels between the GIB and no-GIB groups, and found no notable differences, with reported doses of 0.80 (IQR: 0.60–1.13) *vs.* 0.77 (IQR: 0.50–1.02) ng/ml, respectively.^12^

## Discussion

To the best of our knowledge, this meta-analysis is the first to evaluate the association between digoxin use and GIB occurrence in CF-LVAD-supported patients. Among close to 15,000 patients, the key finding is that digoxin use was associated with a trend toward a lower risk of overall GIB. This association appeared stronger when specifically examining GIB events likely attributable to GIAD.

The study by Jennings *et al.*^23^ had the largest weight in this meta-analysis; however, its broader definition of GIB events—encompassing death, reoperation, rehospitalization, or transfusion—was less specific compared to the definitions used in other studies. This variability in outcome definition may have influenced the pooled results.^23^ Consistent with this, the sensitivity analysis excluding this study showed that digoxin use was associated with a significant reduction in GIB risk.

The overall rate of GIB occurrence reported in our meta-analysis (18.8%) is in line with previously reported rates.^4,6^ Our results included data collected from 2004 to 2019, which implies several key considerations. First, 78% of patients in our meta-analysis had axial CF-LVAD support (HeartMate II), whereas only 22% were supported with centrifugal devices (HeartMate 3 or HeartWare). However, the current standard of care prioritizes the use of the HeartMate 3 device, given its superior outcomes and reduced rates of complications^26^; it is also the only durable LVAD currently broadly available and approved for use. Interestingly, the 5-year analysis of the landmark MOMENTUM 3 (Multicenter Study of MagLev Technology in Patients Undergoing Mechanical Circulatory Support Therapy with HeartMate 3) trial found a difference in GIB risk between centrifugal and axial-flow pump groups, with a relative risk of 0.60 (95% CI: 0.51–0.69), but the event rate in the HeartMate 3 arm remained high (25.2 per 100 patient-years).^27^ In contrast, a real-world prospective registry of 106 CF-LVAD patients (34% with HeartMate 3 and 66% with HeartWare) reported no significant difference in GIB risk between devices (19.4% for Heartmate 3 *vs.* 21.4% for HeartWare over a median follow-up of 1.5 years, *p* = 0.81).^28^ By comparison, the ELEVATE (Evaluating HeartMate 3 with Fully Magnetically Levitated Technology in a Post-Market Approval Setting) registry, which included 463 patients who received the HeartMate 3, reported a GIB event rate of 8 per 100 patient-years within the first two years following LVAD implantation.^29^ Altogether, these findings suggest that the potential protective effect of digoxin against GIB observed with earlier CF devices may remain relevant for patients implanted with a HeartMate 3 device.

Second, most patients were treated with both VKA anticoagulation and antiplatelet therapy. However, the landmark ARIES-HM3 (Aspirin and Hemocompatibility Events With a Left Ventricular Assist Device in Advanced Heart Failure) trial showed that aspirin avoidance reduced nonsurgical bleeding events without increasing the risk of stroke or other thromboembolic events,^30^ including in patients with ischemic cardiomyopathy.^31^ This difference in bleeding risk included GIB (relative risk: 0.61, 95% CI: 0.42–0.87; *p* = 0.007), yet despite this reduction, GIB remained a major concern, with an incidence of 13.1 events per 100 patient-years in the VKA-alone treatment group.^30^ To our knowledge, no study has specifically assessed the association between digoxin use and GIB risk among patients with CF-LVADs treated exclusively with VKA anticoagulants. Emerging evidence on the use of direct anticoagulants in patients with VADs is becoming available, but data on the overall bleeding risk, particularly GIB, associated with this class of anticoagulants remain limited.^32^

Third, none of the studies included in this meta-analysis reported the use of contemporary heart failure guideline-directed medical therapy. Currently, evidence on the use of optimal medical therapy in patients with CF-LVAD support and its relationship with GIB occurrence remains scarce. Emerging evidence suggests that inhibitors of the renin-angiotensin-aldosterone system may have protective effects against GIB in patients supported with VADs.^33^ Blocking activation of the angiotensin-II receptor may inhibit angiogenesis may inhibit angiogenesis by modulating VEGF and Ang-2 pathways, and spironolactone has previously been shown to inhibit angiogenesis in animal models.^33^ Moreover, Jennings *et al.*^23^ reported that the use of beta-blockers was associated with a higher GIB-free survival rate at 5 years of CF-LVAD support. Although the underlying pathophysiological mechanisms for this observation remain unclear, similar results have been reported with beta-blockers in the general population, supporting their potential protective role against GIB in CF-LVAD patients.^34^

Finally, among the four studies included in our meta-analysis, one specifically evaluated the effects of digoxin use on overall mortality and the incidence of right ventricular failure, finding no significant differences between the digoxin and no-digoxin groups for either outcome.^25^ Interestingly, El Rafei *et al.*^24^ reported similar right atrial pressures between the digoxin and no-digoxin groups, supporting the hypothesis that the protective effect of digoxin against GIB, if present, might be mediated through non-hemodynamic mechanisms. Digoxin is known for its narrow therapeutic window, which may pose safety concerns, with higher concentrations potentially associated with a higher risk of mortality.^35^ The most frequent reasons for discontinuing digoxin were worsening renal function and hyperkalemia, highlighting the importance of careful monitoring during therapy to minimize adverse effects. Furthermore, none of the studies provided specific information on digoxin dosages; however, one study reported median digoxin levels within the commonly accepted therapeutic range of 0.5–1 ng/ml for atrial fibrillation management.^12^ Overall, the potential role of digoxin in preventing GIB and its safety profile in patients with CF-LVADs should be carefully evaluated, considering its narrow therapeutic window.^36^

### Limitations

Our study has several limitations. First, the lack of randomized controlled trials comparing digoxin use *versus* non-use among patients supported with CF-LVADs limits this meta-analysis to observational studies, which may be subject to inherent flaws and sources of bias. Second, although availability of baseline population characteristics was an inclusion criterion, the specific variables reported and the level of detail varied considerably between studies. As a result, baseline characteristics could not be uniformly pooled or directly compared across studies. Third, the studies spanned the years 2004 –2019—a period characterized by notable advancements in CF-LVAD technology and heart failure management—potentially resulting in variability in patient care practices, with only a limited number of patients supported by HeartMate 3 CF-LVAD devices. Finally, some degree of patient overlap between studies cannot be entirely excluded, particularly given that one study used data from the INTERMACS registry.^23^

## Conclusions

In our meta-analysis of close to 15,000 patients supported by CF-LVADs, GIB occurred in almost 1 in 5 patients, representing a major source of morbidity. Our results suggest that digoxin use during CF-LVAD support was associated with a trend toward a lower rate of overall GIB occurrence, probably driven by a lower rate of GIB-GIAD occurrence. Further research is needed to validate these findings and guide clinical practice. Specifically, contemporary prospective randomized trials focusing on patients supported by HeartMate 3 devices and treated exclusively by VKAs, are needed.

## Supplementary Material
