# Supplementary material for: Impact of Digoxin Utilization on Gastrointestinal Bleeding in Patients With Continuous-Flow Left Ventricular Assist Devices: A Systematic Review and Meta-Analysis
Source: ASAIO J. 2025 May 18;72(2):137–44. doi: 10.1097/MAT.0000000000002473 (PMC12829496; doi:10.1097/MAT.0000000000002473)
Supplement: Supplementary file 1 [file mat-72-137-s001.pdf]

Supplemental data

Impact of Digoxin Utilization on Gastrointestinal Bleeding in Patients With Continuous-Flow LVADs: A Systematic Review and Meta-Analysis

Matteo Marchetti, Barbara Pitta Gros, Olivier Muller, Clémence Ferlay, Zied Ltaief, Matthias Kirsch, Anna Nowacka, Patrizio Pascale, Philippe Meyer, Patrick Yerly, Pierre Monney, Panagiotis Antiochos, Hicham Skali, Maja Cikes, Roger Hullin, Henri Lu

Supplemental Table 1. Study Quality Assessment Using the Newcastle-Ottawa Scale

Supplemental Table 2. GRADE Evidence Summary Table

Supplemental Table 3. Outcomes and Study Group Definitions

Supplemental Table 1. Study Quality Assessment Using the Newcastle-Ottawa Scale

	**Vukelic et al. (12)**	**Jennings et al. (23)**	**El Rafei et al. (24)**	**Abbasi et al. (25)**
**1. Representativeness of the intervention cohort**<br>a) truly representative of the average CF-LVAD recipient ★<br>b) somewhat representative of the average CF-LVAD recipient ★ (only 1 disease category)<br>c) selected group of patients, e.g. certain insurance coverage, age specific<br>d) no description of the derivation of the cohort	★	★<br>Only patients alive at 3 months post-CF LVAD implantation and receiving both warfarin and antiplatelet therapy were included	★	★
**2. Selection of the non-intervention cohort**<br>a) drawn from the same community as the intervention cohort ★<br>b) drawn from a different source<br>c) no description of the derivation of the non-intervention cohort, or no controls	★	★	★	★
**3. Ascertainment of intervention**<br>a) secure record (e.g., healthcare record) ★<br>b) structured interview ★<br>c) written self-report<br>d) other / no description	★	★	★	★
**4. Demonstration that outcome of interest was not present at start of study**<br>a) yes ★<br>b) no	★	★	★	★
**Comparability (tick one or both boxes, as appropriate)**<br>1. Comparability of cohorts on the basis of the design or analysis<br>a) study controls for age, sex, exposure to the program (survival), disease ★	★	★	★	★

b) study controls for any additional factors (e.g., socioeconomic status, education, geography) ★				
**Outcome (tick one box in each section)**<br>1. Assessment of outcome a) independent blind assessment ★<br>b) record linkage ★<br>c) self-report<br>d) other / no description	★	GIB outcome defined as (1) death, (2) re-operation, (3), hospitalization, (4) transfusion of red blood cells.	★	★
2. Was follow up long enough for outcomes to occur?<br>a) yes, if median duration ≥ 2 months ★<br>b) no, if median duration < 2 months, or unclear	★	★	★	★
3. Adequacy of follow up of cohorts<br>a) complete follow up: all subjects accounted for length of exposure to digoxin (survival bias) ★<br>b) subjects lost to follow up unlikely to introduce bias: number lost ≤ 20%, all ages included, all diseases, or description of those lost suggesting no difference from those followed ★<br>c) follow up rate < 80% (select an adequate %) and no description of those lost, or description suggesting differences from those followed<br>d) no statement	★	★	★	★

Abbreviations: CF-LVAD = continuous flow left ventricular assist device ; GIB = gastrointestinal bleeding.

Supplemental Table 2. GRADE Evidence Summary Table

**Study**	**Risk of bias**	**Inconsistency**	**Indirectness**	**Imprecision**	**Digoxin + (n)**	**Digoxin – (n)**	**Absolute effect (95% CI)**	**Certainty**
**Vukelic et al. (12)**	Low	Not serious	Not serious	Not serious	64	135	HR 0.49 (0.24-0.98)	
**Jennings et al. (23)**	Moderate to serious	Moderate to serious	Moderate to serious	Not serious	2,321	11,402	HR 0.97 (0.87-1.07)	
**El Rafei et al. (24)**	Low	Not serious	Not serious	Not serious	213	436	IRR 0.67 (0.45-0.99)	
**Abbasi et al. (25)**	Low	Not serious	Not serious	Not serious	144	202	HR 0.53 (0.3-0.93)	
**Overall**	**Moderate**	**Moderate**	**Moderate**	**Not serious**	**2,742**	**12,175**	**0.70 (0.49-1.01)**	**+++ : Moderate**
**Sensitivity analysis excluding Jennings et al**	**Low**	**Not serious**	**Not serious**	**Not serious**	**421**	**773**	**0.60 (0.44-0.8)**	**+++: Moderate to high**

Outcome: GIB occurrence among patients with CF-LVAD support using digoxin vs. non-using digoxin.
Abbreviations: CF-LVAD = continuous flow left ventricular assist device; CI = confidence interval; GIB = gastrointestinal bleeding; GRADE = Grading of Recommendations Assessment, Development, and Evaluation; HR = hazard ratio; IRR = incidence rate ratio.

Supplemental Table 3. Outcomes and Study Group Definitions

	**Vukelic et al. (12)**	**Jennings et al. (23)**	**El Rafei et al. (24)**	**Abbasi et al. (25)**
**Definition of GIB**	Clinical evidence of bleeding (haematochezia, hematemesis, melena, positive faecal-occult blood test), along with a haemoglobin decrease of at least 1 g/dl.	Suspected internal or external bleeding that resulted in one more of the following: (1) death, (2) re-operation, (3) hospitalization, (4) red blood cell transfusion.	Clinical evidence of bleeding (haematochezia, hematemesis, melena, positive faecal-occult blood test), along with a haemoglobin decrease of at least 1 g/dl.	Clinical evidence of bleeding (haematochezia, hematemesis, melena, positive faecal-occult blood test), along with a haemoglobin decrease of at least 1 g/dl.
**Digoxin group definition**	Patients who received digoxin for at least 7 days after CF-LVAD implantation and were still on the drug at discharge.	Digoxin prescription at 3 months post CF-LVAD implantation.	Digoxin prescribed either at discharge or within 3 months post-discharge, following CF-LVAD implantation.	Digoxin use for at least 30 days after CF-LVAD implantation.

Abbreviations: CF-LVAD = continuous flow left ventricular assist device ; GIB = gastrointestinal bleeding.
